# Examining transitional care for patients and caregivers: Lessons from a Brazilian public university hospital

**DOI:** 10.1016/j.clinsp.2025.100756

**Published:** 2025-08-21

**Authors:** Mariana Taddeo Dal Medico, Larissa Vidal, Ivaldo Olimpio da Silva, Thales Chelala Toledo, Gabriela Alves de Oliveira Hidalgo, Eduardo da Silva Santocchi, Douglas Henrique Crispim

**Affiliations:** a Programa de Estudos Avançados em Administração Hospitalar e Sistemas de Saúde do Hospital das Clínicas da Faculdade de Medicina da Universidade de São Paulo (HC-FMUSP), São Paulo, SP, Brazil; b Instituto Perdizes (IPER), Hospital das Clínicas da Faculdade de Medicina da Universidade de São Paulo (FMUSP), São Paulo, SP, Brazil; c Faculdade de Medicina da Universidade de São Paulo (FMUSP), São Paulo, SP, Brazil

**Keywords:** Transitional care, Patient discharge, Continuity of patient care, Process assessment, Hospital to home transition

## Abstract

**Objective:**

This study aimed to assess patient and caregiver perceptions following discharge from a hospital transitional care unit in a public university hospital in Brazil. *Method:* This cross-sectional study collected data through telephone contact using the adapted Brazilian version of the Care Transitions Measure (CTM-15 Brazil).

**Results:**

The present study showed better results in addressing key patient preferences and challenges in the care plan. The main results indicated that patients with chronic diseases or injuries had higher approval of the transitional care service. The main findings indicated that patients with chronic diseases or injuries had higher approval of the transitional care service. Additionally, a significant correlation was found between age and total score (positive) and age and care plan (negative).

**Conclusions:**

Hospital transitional care units are crucial for reintegrating patients after hospitalization. Evaluating services with validated scales, like the CTM-15 Brazil, helps identify improvement areas and track outcomes.

## Highlights

• Examined patient and caregiver perceptions post-hospital discharge in Brazil.
• Employed CTM-15 Brazil for data collection on transitional care quality.
• Patients with chronic illnesses showed higher satisfaction with transitional care.
• Hospital transitional care units play a vital role in patient reintegration.
• Emphasized the importance of personalized care plans for enhancing outcomes

## Introduction

In a country undergoing demographic and epidemiological transition, with an increase in chronic diseases occupying hospital beds,^1^ dialogue between different levels of care is essential. While hospitals respond to the demands of acute conditions and events, it is not the most suitable for managing individuals with chronic diseases.^2^

Moreover, the complexity of the healthcare system hinders the transfer of care to primary health care after patients are discharged from hospital care. Patients often cannot access primary health care, despite it being the most appropriate for chronic conditions, leading to patients feeling abandoned after leaving the healthcare facility.^2^^,^^3^

To prevent this feeling of abandonment, the transitional care was conceived, which is defined as a set of actions aimed at ensuring the coordination and continuity of healthcare when transferring a patient from one setting to another, such as between healthcare facilities, between levels of healthcare attention, between the hospital and home, or between the hospital and another institution.^4^ The literature indicates that transitional care leads to cost reduction and hospital readmissions, as well as an increase in patient quality of life and satisfaction with discharge.^5^^,^^6^

The Complex of the Hospital das Clínicas da Faculdade de Medicina da Universidade de São Paulo (HC-FMUSP) is the largest hospital complex in Latin America, comprising nine large hospitals, each dedicated to specific areas. One of them is Instituto Perdizes (IPER), which, since May 2023, has been responsible for the transitional care unit, with 90 dedicated beds, increasing turnover rates in other hospitals within the complex and ensuring access for other users of Brazil's public healthcare system.^7^

The IPER care model includes active patient recruitment from other hospitals, development of individualized multidisciplinary therapeutic plans, early approach for discharge planning, caregiver training, and integration with public service networks based on the patient's region of residence. The unit operates with an average length of stay of 20 days.

The aim of this study is to measure the quality of transitional care services from the perspective of patients discharged from the hospital to home and to identify patient characteristics associated with perceived quality.

## Materials and methods

A cross-sectional observational and epidemiological study was conducted at the care transition unit of IPER of HC-FMUSP. Approved by the Ethics Committee for Human Research Protocols of the Hospital das Clínicas da Faculdade de Medicina da Universidade de São Paulo n 76281223.2.0000.0068. All participants signed the free and informed consent form through an electronic form. As a cross-sectional observational study, this study follows the STROBE statement.

The study population consisted of patients who were admitted to the transitional care unit and were discharged home between May 2023 and February 2024. Patients who died after discharge and those transferred to other institutions were excluded.

Data collection was conducted between March and April 2024 through telephone contact, applying the adapted Brazilian version of the Care Transitions Measure (CTM-15 Brazil),^8^ designed to assess the quality of transitional care from the patients and their caregivers’ perspective.^9^ It has been used in healthcare services in various countries and has proven to be reliable, accurate, and valid.^10^

The CTM-15 comprises 15 statements with five response options, recommended by the instrument authors to be scored as follows: “strongly disagree” = 1-point; “disagree” = 2-points; “agree” = 3-points; “strongly agree” = 4-points, while the neutral option “don't know/I don't remember/not applicable” does not receive a score as it is not included in the calculation of the final score. However, it is differentially encoded to verify the percentage of this response across instrument items.^8^^,^^9^

The CTM-15 items are grouped into four factors considered essential for measuring care transition: Health management preparation (1), Medications understanding (2), Important preferences (3), and Care plan (4), as shown in Table 1.

**Table 1:** Identification of the items in the instrument Care Transitions Measure (CTM-15), São Paulo, SP, Brazil, 2024.

CTM-15 item	CTM-15 factor
1. Agreed health goals and means.	3
2. Preferences for deciding health needs.	3
3. Preferences of deciding where needs are met.	3
4. Had information needed for self-care.	1
5. Understand how to manage health.	1
6. Understand warning signs and symptoms.	1
7. Had a written care plan.	4
8. Understand what makes me better or worse.	1
9. Understand things I was responsible for.	1
10. Confident I knew what to do.	1
11. Confident I could do what needed.	1
12. Had written list of appointments and tests.	4
13. Understand purpose of medications.	2
14. Understand how to take medications.	2
15. Understand side effects of medications.	2

Three calls were made to each patient using the phone number listed in the electronic health record. Patients who did not answer the calls were excluded from the study. If the patient was unable to respond to the instrument, caregivers who had followed the instructions provided during the hospitalization and discharge period were allowed to respond.^8^

Although the creators of the CTM-15 recommend applying the instrument within one to four weeks after discharge, in this study, this period was longer for most patients, as the authors assessed the service from the beginning of its implementation, which may introduce recall bias.^10^

The independent variables were also extracted from the electronic health record: gender, age, city of residence, marital status, disease characteristic, and length of stay. And the study's dependent variables were calculated from the means of the total scale and factors of the CTM-15 Brazil, which were transformed into scores from 0 to 100 using the formula suggested by the CTM-15 authors.^11^ Thus, standardized scores of the care transition scale were obtained, evaluated globally, and through factors 1, 2, 3, and 4.

### Statistical analysis

Descriptive analysis of the data was applied with calculation of absolute frequency (n) and percentage (%) for categorical variables, and calculation of measures of central tendency and dispersion (mean, median, standard deviation, minimum, and maximum values) for continuous variables.

In the analytical plan, nonparametric Spearman's correlation tests and Wilcoxon-Mann-Whitney tests were chosen. The significance level used as the criterion for acceptance or rejection in statistical tests was 5 % (p ≤ 0.05), and the STATA version 16 program was used.

## Results

Out of 242 eligible patients, 38 declined to participate, 14 had incorrect phone numbers in the registry, and 82 could not be reached after three attempts. As a result, the study included 34 patients and 74 caregivers, totaling 108 participants. Whose characteristics are presented in Table 2.

**Table 2:** Characterization of the participants included in the research, São Paulo, SP, Brazil, 2024.

Participants’ Characteristics	Mean (SD), Min‒Max or n (%)
**Gender**	
Male	56 (51.85)
Female	52 (48.15)
**Marital status**	
Married	43 (39.81)
Single	47 (43.52)
Widowed	8 (7.41)
Divorced	10 (9.26)
**City of residence**	
Same city of the hospital	65 (60.18)
Other cities	43 (39.82)
**Disease characteristic**	
Chronic disease or injury	61 (56.48)
Acute disease	47 (43.52)
**Age, mean (SD), min-max**	56.35 (16.73), 21‒88
**Length of stay, mean (SD), min‒max**	19.10 (12.41), 4‒91

Regarding the CTM-15 Brazil, the average total score identified in this research was 64.01. The factor with the highest score was factor 3 (important preferences), and the factor with the lowest score was factor 4 (care plan), as shown in Table 3.

**Table 3:** Mean of care transition quality according with total score and CTM-15 factors, São Paulo, SP, Brazil, 2024.

Scores	Mean ± SD
CTM Total Score	64.01 ± 17.22
CTM-15 factors	
Factor 1 – Health management preparation	64.84 ± 18.18
Factor 2 – Medications understanding	63.75 ± 21.04
Factor 3 – Important preferences	66.45 ± 20.81
Factor 4 – Care plan	57.84 ± 25.98

Table 4 presents the mean and standard deviation obtained for each item of the instrument.

**Table 4:** Mean of care transition quality according with CTM-15 items, São Paulo, SP, Brazil, 2024.

CTM-15 items	CTM-15 factors	Mean ± SD
14. Understand how to take medications.	2	71.38 ± 19.75
4. Had information needed for self-care.	1	70.06 ± 22.29
1. Agreed health goals and means.	3	69.45 ± 21.43
13. Understand purpose of medications.	2	69.16 ± 22.75
5. Understand how to manage health.	1	68.21 ± 22.96
8. Understand what makes me better or worse.	1	66.67 ± 22.78
9. Understand things I was responsible for.	1	66.67 ± 20.98
3. Preferences of deciding where needs are met.	3	65.74 ± 25.56
2. Preferences for deciding health needs.	3	64.51 ± 26.29
11. Confident I could do what needed.	1	60.44 ± 27.52
6. Understand warning signs and symptoms.	1	60.32 ± 22.69
12. Had written list of appointments and tests.	4	60.12 ± 30.52
10. Confident I knew what to do.	1	60.12 ± 27.26
7. Had a written care plan.	4	56.31 ± 29.16
15. Understand side effects of medications.	2	50.64 ± 32.51

Table 5 shows the correlation between the total scores and the factor scores of the CTM-15 Brazil and the independent variables of the study. There was a statistically significant correlation between three of them. Additionally, there is no statistically significant difference between caregivers and patients who responded to the CTM-15 Brazil.

**Table 5:** Association and correlation between the CTM-15 total score, score per factor and independent variables, São Paulo, SP, Brazil, 2024.

Variables	Total Score	Factor 1	Factor 2	Factor 3	Factor 4
**Age**^b^	0.2064	−0.0885	−0.1045	−0.0275	−0.2512
p^c^	**0.0321**	0.3625	0.282	0.7778	**0.0091**
**Gender**^a^					
Male	60.65 ± 17.67	67.13 ± 17.88	61.32 ± 20.56	68.31 ± 20.64	59.12 ± 23.47
Female	67.62 ± 16.11	62.31 ± 18.34	66.44 ± 21.45	64.40 ± 21.03	56.46 ± 28.63
p^d^	0.0636	0.2255	0.4717	0.3968	0.6274
**City of residence**^a^					
Same city of the hospital	62.76 ± 19.25	65.16 ± 19.15	64.24 ± 21.91	65.28 ± 19.14	57.41 ± 26.40
Other cities	65.89 ± 13.59	65.95 ± 16.63	62.96 ± 19.80	68.38 ± 23.44	58.55 ± 25.61
p^d^	0.2994	0.6488	0.2249	0.8519	0.463
**Disease characteristic**^a^					
Chronic disease or injury	64.58 ± 18.69	64.77 ± 18.84	61.99 ± 22.90	65.89 ± 23.37	57.14 ± 25.59
Acute disease	63.26 ± 15.26	64.92 ± 17.52	65.94 ± 18.50	67.75 ± 17.36	58.69 ± 26.70
p^d^	**0.0417**	0.9568	0.5523	0.5604	0.7976
**Length of stay**^a^					
≤ 20 days	62.64 ± 16.83	65.29 ± 18.65	63.39 ± 21.02	65.94 ± 21.46	56.83 ± 26.77
> 20 days	66.07 ± 17.78	64.17 ± 17.67	64.29 ± 21.33	67.20 ± 20.08	59.95 ± 25.01
p^d^	0.6989	0.9717	0.5386	0.3113	0.289
**Marital status**^a^					
Married	67.41 ± 15.29	67.86 ± 15.44	67.48 ± 18.15	69.11 ± 19.80	58.54 ± 24.47
Others	61.75 ± 18.14	62.84 ± 19.65	61.29 ± 22.56	64.70 ± 21.44	57.38 ± 27.14
p^d^	0.1976	0.3216	0.2094	0.4124	0.8575

a Values expressed as mean value ± standard deviation.

b Values expressed as correlation coefficients.

c Using nonparametric Spearman’s correlation.

d Using Wilcoxon-Mann-Whitney test.

## Discussion

In relation to the total score, the authors establish that the higher the score, the better the transitional care. In this study, the average total score was 64.01 on the CTM-15 Brazil.^8^ This is interesting because this score is a metric comparable to other healthcare settings. Another study conducted in a public hospital in the state of São Paulo obtained an average score of 68.59,^10^ while in southern Brazil, other studies in adults found total score values of 69.5,^12^ 74.7,^13^ and 76.8.^14^ Similarly, in the United States of America, a study using the CTM-15 with patients who had discharge preparation obtained 74.7.^15^ Studies conducted in Sweden obtained total scores of 61.8^16^ and 65.7,^17^ and in Japan 66.3,^18^ presenting values closer to those of this study. There are studies on the acquiescence bias of the CTM-15, which may contribute to positive study results.^19^

Many factors can contribute to this total score, one of them being the fact that the patients evaluated in this sample range from the first patients treated in the unit, which had a phase of implementation and adaptation to the new care transition model. Another factor to be considered is the profile of patients treated by IPER, which is complex, as patients come from hospitals with different clinical profiles, ranging from patients with cardiac diseases to severe injury.^20^

The highest score found was for factor 3 (important preferences) with a value of 66.45, indicating that professionals are committed to considering patients' health needs and preferences. This commitment is manifested in institutional actions such as the implementation of extended visits, adaptation of diet menus according to patients' preferences, and the availability of a 24-hour companion. The institution's culture, from the beginning, has focused on developing genuine and empathetic relationships with patients and caregivers, providing support and training to them, in addition to excellent clinical care, which contributes to factor 3 receiving the highest score.

On the other hand, the lowest score found in this research (57.84) was for factor 4 (care plan), highlighting the importance of improving the quality of the written care plan and the consultations and tests to be carried out after discharge, which, when meeting the patient's clinical and social needs, can promote appropriate use of health resources and services.^21^ Some factors contributing to this score include Brazil's lack of uniformity in scheduling exams and consultations, despite efforts to implement this, and limitations regarding hospital integration with primary health care, which leads discharged patients to face difficulties in finding medications and consultations near their homes, as transitional care involves both hospital and municipal care.^5^^,^^22^ Despite the efforts the service has made to build a model that adapts to different realities, educational levels, and income, which is easy to understand and not forgotten after discharge, it still faces various challenges due to the heterogeneity of patients from a cultural standpoint.

Item 15, which addresses patients' understanding of medication side effects, had the highest disagreement (40.7 %), similar to what was found in other studies conducted in Brazil, which also presented this item with the lowest scores among the studies.^12^^,^^13^ In this item, the authors observe the importance of regular medication guidance during hospitalization, and considering the high prevalence of people using multiple medications, written guidance may become necessary.^23^ These findings highlight the need for institutional strategies aimed at strengthening communication about medications during discharge planning. Public hospitals could benefit from implementing standardized discharge protocols that include written and verbal guidance about potential side effects, especially for patients with polypharmacy, as a means to improve transitional care and patient safety.

Regarding the analysis of the correlation between variables, there was a statistically significant correlation between age and total score, being positive, and age and factor 4, being negative. Although statistically significant, these relationships did not demonstrate a clear connection between patients' age and a more positive or negative perception as measured by the scale. One hypothesis proposed is that older patients often present with more complex health conditions, which may require greater planning and care from the team, increasing their perception of value regarding the service received. On the other hand, the negative correlation between age and factor 4 suggests that younger individuals may find it easier to understand the care plan outlined for them. Another possibility is that generational differences in communication styles influence perceptions, as younger individuals may prefer more agile approaches than older ones. These findings reinforce the importance of tailoring discharge communication strategies to different age groups and ensuring that the care plan is not only understood but also integrated into the follow-up by primary health care services in the public health system.

Furthermore, there is a statistically significant difference between the scores given for care transition between the groups with chronic disease or injury and the group that was hospitalized for acute disease treatment, with the score being higher in the group of patients hospitalized with chronic disease or injury and lower in the group hospitalized for acute illness treatment (p = 0.0417). Considering the context of the healthcare network, this difference is interesting because it apparently demonstrates the need for the first group to have the bridging role performed by transitional care, as indicated by different network models.^2^ Cases of acute disease involved patients who were hospitalized for the completion of medications such as antibiotics, anticoagulants, and others, largely comprising individuals with greater functionality and independence who, generally, will spend less time with healthcare professionals by their side during their hospitalization. Patients with more complex needs had a more positive perception of the service, reinforcing the importance that transitional care plays in maintaining the quality of life of these individuals and reinforcing the perception of the role of transitional care units in safe discharge.^24^ In a unit where colleagues are encouraged to develop a more empathetic relationship with patients, this increased contact can result in better memories and positive perceptions of hospitalization.

A limitation of this study is the time interval between the interview and the event. Although the recommendation is to apply the instrument within one to four weeks after the transition, in the present study, it was applied with a median of three months after hospital discharge.^8^^,^^9^ This extended interval may introduce recall bias, a type of systematic error that occurs when participants do not accurately remember past events or experiences. This bias can lead to misclassification of experiences or outcomes, potentially affecting the reliability of the findings. Therefore, this recall bias should be acknowledged as a limiting factor in the investigative process. Furthermore, the authors recognize that the non-participation rate may have introduced selection bias, which limits the generalizability of the findings, as the participants included in the study may not fully represent the broader population of eligible patients.

Despite this, the study is important as it analyzes the implementation phase. The fact of contacting patients late reinforces the need to maintain the literature recommendations to make contact as early as possible, avoiding the loss of important information and memories of hospitalization and discharge by the interviewee. Additionally, patients' questions and uncertainties, along with assistance in navigating the complex healthcare service network, can be addressed after hospital discharge. The presence of a “monitoring nurse” can serve as a starting point for accessing healthcare services, assisting primary care professionals in better understanding patients' real needs, and consequently reducing the need for return to more specialized hospital services.^25^^,^^26^

## Conclusions

Hospital transitional care units play an important role in reintegrating patients back home after hospitalization. Evaluating services using validated scales helps understand real improvement demands and track outcomes. Assessing the quality of transitional care services in this study through the CTM-15 Brazil showed better results in the field of important preferences and worse in the care plan field. Age and the presence of chronic disease or injury were statistically significant variables, the latter suggesting that patients with higher care demands had greater approval of the service by themselves and their families.

## CRediT authorship contribution statement

**Mariana Taddeo Dal Medico:** Conceptualization, Investigation, Formal analysis, Project administration, Writing – original draft. **Larissa Vidal:** Investigation. **Ivaldo Olimpio da Silva:** Software, Formal analysis. **Thales Chelala Toledo:** Resources. **Gabriela Alves de Oliveira Hidalgo:** Writing – review & editing. **Eduardo da Silva Santocchi:** Resources, Writing – review & editing. **Douglas Henrique Crispim:** Conceptualization, Supervision, Writing – review & editing.

## Conflicts of interest

The authors declare no conflicts of interest.
